# Cadmium toxicity in plants: from transport to tolerance mechanisms

**DOI:** 10.1080/15592324.2025.2544316

**Published:** 2025-08-22

**Authors:** Yutong Hu, Rui He, Xueyi Mu, Yuanhang Zhou, Xiaodong Li, Hao Wang, Wang Xing, Dali Liu

**Affiliations:** a National Beet Medium-term Gene Bank, Heilongjiang University, Harbin, Heilongjiang, China; b Key Laboratory of Sugar Beet Genetics and Breeding, Heilongjiang Province Common College/College of Advanced Agriculture and Ecological Environment, Heilongjiang University, Harbin, Heilongjiang, China; c Manas Agricultural Experimental Station, Xinjiang Academy of Agricultural Sciences, Changji, China; d Inner Mongolia Key Laboratory of Sugar Beet Genetics and Germplasm Enhancement, Inner Mongolia Academy of Agricultural and Animal Husbandry Sciences, Huhhot, China

**Keywords:** Cadmium toxicity, tolerance, antioxidant defense, chelation, transport

## Abstract

Cadmium (Cd), a non-essential heavy metal, induces severe phytotoxicity through oxidative stress and cellular homeostasis disruption. Chronic Cd exposure inhibits plant growth via leaf chlorosis, stunted stem elongation, and impaired root architecture, while disrupting physiological functions through chlorophyll degradation, membrane peroxidation, and antioxidant system collapse. This review systematically investigates plant adaptive responses to Cd stress. It examines the processes of Cd uptake pathways, translocation dynamics, physiological toxicity, and molecular defense mechanisms. Key findings highlight two main protective strategies: avoidance mechanisms involving root secretion regulation, cellular compartmentalization, efflux transport, and the other through chelation, antioxidant systems, and phytohormonal regulation in tolerance mechanisms. A particular emphasis is placed on the coordinated actions between metal-chelating compounds (including PCs, MTs, and MTPs) and both enzymatic (SOD, CAT) and non-enzymatic antioxidants. These insights advance the theoretical framework for plant Cd resistance and inform innovative implications for developing effective remediation approaches.

## 1. Introduction

Cadmium (Cd), a consistent phytotoxic heavy metal, disrupts plant growth through various physiological impairments. The severity of Cd toxicity exhibits significant dose-dependency and species-specificity. For instance, studies indicate that a Cd^2+^ concentration greater than 10 µmol/L significantly inhibited the growth of *Arabidopsis thaliana* L., as evidenced by reduced root elongation and wilting.^1^ In contrast, known hyperaccumulator plants, such as *Sorghum bicolor* L., can tolerate aqueous Cd^2+^ concentrations exceeding 100 µmol/L while actively accumulating over 50 mg/kg of dry weight in their young tissue.^2^ Generally, at toxic concentrations, Cd induces chlorosis, growth inhibition, and root architecture alterations via oxidative damage to chlorophyll and membrane integrity.^3^ In response to Cd stress, plants employ multiple detoxification strategies, including perception, chelation, vacuolar compartmentalization, enzymatic ROS scavenging, and efflux transport, which collectively form a coordinated defense network.^4^ Understanding these processes is essential for developing genetically engineered hyperaccumulators, thereby enhancing phytoremediation technologies for soil decontamination and ecological restoration. Progress in understanding Cd accumulation and tolerance mechanisms across scales, from whole-plant to subcellular levels, has been significantly advanced by analytical techniques such as ICP-MS (Inductively Coupled Plasma Mass Spectrometry),^5^ µ-XRF (Micro X-ray Fluorescence),^6^ XANES (X-ray Absorption Near Edge Structure),^7^ and emerging methods like LA-ICP-MS (Laser Ablation ICP-MS) now enabling in situ Cd mapping at subcellular resolution (1 µm).^8^

## 2. Cadmium uptake, transport, and accumulation in plants

The distribution of Cd^2+^ varies across different plant tissues and organs, which is closely associated with the plant’s tolerance to Cd^2+.9^ After the uptake by root systems, Cd^2+^ is primarily immobilized in the cell wall or vacuoles, while a portion is transported to the xylem for further distribution and translocation. Additionally, some metal ions are also transported into the phloem. This differential distribution and compartmentalization play a key role in the plant’s ability to manage and mitigate Cd tolerance.^10^

### 2.1. Uptake of Cd^2+^ by plant roots

Plant roots primarily absorb Cd^2+^ from contaminated soil through two distinct mechanisms regulated by both soil factors and plant genetic characteristics. The absorption efficiency depends on soil Cd concentration, pH levels, plant species, and cultivation conditions.^11^ Subsequent translocation and distribution of Cd within plant tissues are governed by complex physiological regulation systems.

**Figure 1.**
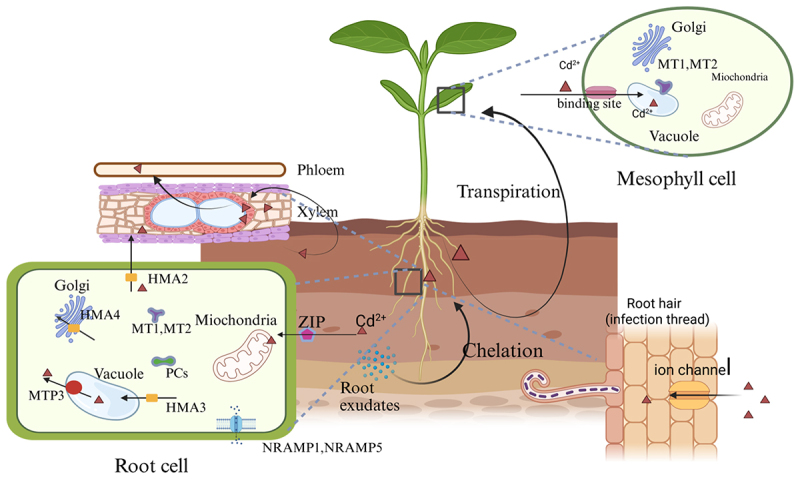
Cadmium uptake and transport in plants. MT1, MT2 (metallothionein 1 and 2); HMA2, HMA3, HMA4 (heavy metal ATPase 2, 3 and 4); PCs (phytochelatins); MTP3 (metal tolerance protein 3). NRAMP1, NRAMP5 (natural resistance-associated macrophage protein 1 and 5). (mapping with BioRender).

The first uptake mechanism involves rhizospheric chelation facilitated by root exudates (Figure 1).^12^ These organic compounds, particularly low molecular weight organic acids (LMWOA), enhance heavy metal bioavailability through ligand complexation, thereby promoting metal absorption and accumulation in roots.^13^ Liu et al.^14^ demonstrated that *Oryza sativa* L. roots modulate LMWOA secretion in response to soil Cd^2+^ concentrations and genotypic variations, suggesting this process constitutes a critical determinant of interspecific differences in Cd uptake efficiency. Chen et al.^15^ further elucidated the concentration-dependent effects of root exudates, reporting optimal Cd removal efficiency at 100 mmol/L citric acid, whereas low concentrations of glycine and maltose enhanced plant biomass remediation performance.

The second mechanism relies on transmembrane transport through specialized ion channels. Cd^2+^ ions initially penetrate root hair cells via specific transporters in semipermeable plasma membranes, then migrate into cortical tissues through symplastic pathways.^16^ This transport structure highlights the importance of root structure in regulating metal uptake. For instance, *Zea mays* L. root architecture demonstrates significant correlations with Cd absorption capacity under varying cultivation conditions, particularly in relation to water availability and nutrient status.^17^ These processes work together under precise physiological regulation to control Cd uptake while preserving essential metabolic functions. The contribution of each pathway varies with plant species and environmental factors, highlighting the complexity of metal homeostasis in plants.

### 2.2. Intracellular and systemic transport of Cd^2+^

Following root absorption, Cd^2+^ undergoes compartmentalized transport through two vascular systems: predominant xylem-mediated distribution and limited phloem-assisted translocation (Figure 1). Variations in metal transport capacity among species are well documented, with hyperaccumulators exhibiting specialized mechanisms.^18^ *Noccaea caerulescens* L. (a Cd-hyperaccumulator species) develops a highly selective Cd transport system in the plasma membrane of root cells, resulting in exceptional Cd accumulation.^19^ However, Yang et al.^20^ reported differential Cd allocation patterns: preferential shoot translocation in *Brassica oleracea* L. and *Zea mays* L., versus root sequestration in *Lolium perenne* L.

This metal partitioning is governed by coordinated gene networks regulating cellular detoxification and systemic distribution.^21^ ZRT/IRT-like proteins (ZIPs) mediate nonspecific Cd^2+^ uptake via transporters (e.g., IRT1), contributing to rhizospheric accumulation.^22^ Cation diffusion facilitators (CDFs) facilitate vacuolar sequestration via antiporters (e.g., *MTP3* and *MTP4*), thereby reducing cytosolic toxicity. *MTP3* is specifically localized to the vacuolar membrane for Cd compartmentalization^23^ Natural resistance-associated macrophage proteins (NRAMPs) enabling long-distance transport through symplastic loading, particularly *NRAMP1* and *NRAMP5* mediated root-to-shoot translocation. *NRAMP5* also plays a critical role in Mn/Cd cotransport.^24^ Heavy metal ATPases (*HMA2*, *HMA3*, and *HMA4*) contribute to xylem loading and systemic distribution.^25^ *HMA3* sequesters Cd into vacuoles alongside phytochelatins (PCs), forming Cd-PC complexes for detoxification. These transporter systems exhibit functional specialization and regulatory interactions.^26^ Metallothioneins (*MT1* and *MT2*) provide additional cytosolic metal buffering capacity, particularly in roots and shoots.^27^ Yang et al.^28^ observed a metabolic balance between Cd uptake via ZIP transporters and efflux via CDF/HMA transporters, with NRAMP overexpression disrupting homeostasis through the saturation of ion channels. The dynamic equilibrium among these transporters, including the Golgi-localized *HMA4* and the vacuolar *MTP3*, ultimately determines the specific Cd allocation patterns and tolerance thresholds of plants.

### 2.3. Tissue-specific accumulation of Cd^2+^

Following xylem-mediated translocation, Cd preferentially accumulates in aerial tissues through transpiration. Foliar sequestration primarily occurs in vacuolar via coordinated action of heavy metal transporters and high-affinity binding sites on the cell membrane.^29^ The Cd content in different plant tissues and its transfer capacity in the rhizosphere vary significantly. *Brassica juncea* L. exhibits four- to sixfold higher root-to-shoot Cd ratios^30^; however, in *Triticum aestivum* L., Cd accumulation in the root system decreases as Cd levels in seed grains increase, with a corresponding rise in accumulation in leaves and roots as seed Cd content declines.^31^ Phylogenetic analysis reveals distinct Cd accumulation strategies across plant families. Fabaceae display intrinsically low Cd uptake, contrasting with hyperaccumulators of *Brassicaceae* L. and *Chenopodiaceae* L. and intermediate accumulators of *Poaceae* L. and *Liliaceae* L.^32^ These findings were further supported by experiments with *Asteraceae* L. and *Apiaceae* L. Under 40 mg/kg Cd exposure, *Brassica oleracea* ‘KG’ accumulated 18.84 mg/kg DW in shoots 260-fold higher than control.^33^ Similarly, under high Cd stress, *Spinacia oleracea* L. accumulated much higher levels of Cd than *Raphanus sativus* L. and *Brassica campestris* L., reaching a total of 16.35 µmol/L.^34^ Different plants exhibit varying abilities to absorb and accumulate Cd, with distinct absorption capacities across different plant parts. However, when Cd concentrations exceed the plants’ tolerance limits, it can severely inhibit their growth and development.^35^

## 3. Toxic effects of cadmium on plants

Cd exposure triggers complex physiological disruptions in plants, primarily resulting in growth retardation, morphological abnormalities, photosynthetic inhibition, and oxidative damage.

### 3.1. Growth inhibition and morphological damage

Heavy metal stress inhibits root growth, with Cd binding to cell wall pectin and reducing cell wall elasticity, thereby destroying root nodules.^36^ It was found that the treatment of *Glycine max* L. with 40 µmol/L CdCl_2_ not only significantly reduced the dry and fresh weights of the whole plant but also reduced the root length and related.^37^ Zhou et al.^38^ further identified a protective film in *Oryza sativa* L. roots under Cd stress, which partially immobilized Cd but hindered oxygen diffusion, leading to programmed cell death in the root tip. Above-ground development is also severely impaired, as Cd inhibits the activity of the apical meristematic tissue in the stem, leading to a loss of apical dominance.^39^ Marina et al.^40^ found that *Solanum lycopersicum* L. seedlings grown in soil contaminated with 10 mg/kg Cd experienced a 38% reduction in height, with internode shortening closely related to deformations of xylem conduits. Similarly, in *Triticum aestivum* L., Cd accumulation in flag leaf (up to 5 mg/kg) resulted in disorganized chloroplast arrangement, a 22% reduction in chloroplast thickness, and a significant decrease in leaf area.^41^ In *Zea mays* L, Cd concentrations above 50 mg/L severely inhibited seed germination and seedling growth, with visible symptoms, such as browning of root tips and degeneration of lateral roots.^42^

### 3.2. Effects on plant photosynthesis and nutrients

Cd exposure induces systemic dysfunction in photosynthetic processes through structural and biochemical disruptions.^43^ The metal directly suppresses chlorophyll biosynthesis and photosynthetic capacity.^44^ Babar et al.^45^ reported that Cd treatment during the tillering stage in *Oryza sativa* L. led to a significant decrease in chlorophyll content, negatively impacting photosynthesis, growth, and development. Cd stress also induces ultrastructural damage in chloroplasts. Transmission electron microscopy (TEM) revealed ruptured chloroplast envelopes, tripled starch grain volume, and disorganized thylakoid membranes in *Zea mays L*. leaves.^46,47^ μ-XRF further localized Cd accumulation in vascular bundle sheath cells, disrupting the transport of substances within the chloroplast.^48^ High Cd^2+^ concentrations in soil inhibit nutrient uptake by plants, either due to cation competition or direct impacts on root growth.^49^ Yang et al.^50^ found that Cd toxicity reduced Cu, Zn, Fe, Mn, Ca, and Mg in *Lolium perenne* L. In *Brassica juncea* L., Cd affected P, K, Ca, Fe, and Zn in roots, and P, K, Ca, and Cu in aerial parts.^51^

### 3.3. Impact on oxidative stress

Cadmium disrupts cellular redox equilibrium by catalyzing Fenton-type reactions through Fe^2+^/Cu^2+^ substitution, sustaining reactive oxygen species (ROS) overproduction.^52^ In *Triticum aestivum* L. seedlings, 3.3–10 mg/kg soil Cd marks the transition from compensated to decompensated oxidative states, with superoxide dismutase (SOD), catalase (CAT), ascorbate peroxidase (APX), and glutathione reductase (GR) activities maintaining homeostasis below 3.3 mg/kg but showing variability at higher concentrations.^53^ This biphasic response pattern extends to membrane integrity indicators, as evidenced in *Brassica napus* L. leaves, where 100 μmol/L Cd^2+^ exposure for 24 h elevated malondialdehyde (MDA) levels 3.5-fold and increased electrolyte leakage by 80%.^54^ Plants activate their antioxidant enzyme systems to combat oxidative damage. Under Cd stress, *Nicotiana tabacum* L. SOD activity increased by 1.8-fold at low concentrations (25 µmol/L) but was inhibited at high concentrations (100 µmol/L) due to protein denaturation.^55^ Similarly, in *Arabidopsis thaliana* L., Cd triggered a 2.3-fold increase in the expression of GSH1, a key gene involved in glutathione (GSH) synthesis. However, prolonged exposure decreased the GSH/GSGG ratio, leading to a breakdown in redox homeostasis.^56^

## 4. Mechanisms of Cd^2+^ perception and detoxification in plants

### 4.1. Cd^2+^ perception

Plants have evolved specific mechanisms to sense cadmium ions that initiate detoxification responses. The perception process begins with extracellular binding: Cd^2+^ initially becomes immobilized by binding to the carboxyl groups of pectin and hemicellulose in the root cell wall. This process is enhanced by the negative charge effect and the activation of pectin methyl esterase (PME).^36^ Upon entering cells, Cd^2+^ exploits structural similarities to essential metals (ionic radius comparable to Fe^2+^, Zn^2+^, and Ca^2+^) to hijack metal transporters. It competitively occupies an influx of transporters including IRT1, ZIP, and NRAMP.^22^ For example, AtIRT1 deletion resulted in a 70% reduction in Cd^2+^ uptake without affecting Zn^2+^ transport.^57^ Intracellularly, Cd^2+^ directly disrupts calcium signaling by interfering with Ca^2+^ channels and mimics Ca^2+^ signals, activating calmodulin (CaM) and calcium-dependent protein kinases (CDPKs).^52^ Cd^2+^ also functions as a molecular “decoy” due to its high affinity for thiol groups (−SH). It binds to glutathione (GSH) and cysteine residues in proteins (e.g., transcription factors, enzymes), altering their conformation and activity. This thiol-sensing mechanism directly induces expression of detoxification genes like PCs and MTs.^58^

### 4.2. Integrated detoxification mechanisms

**Figure 2.**
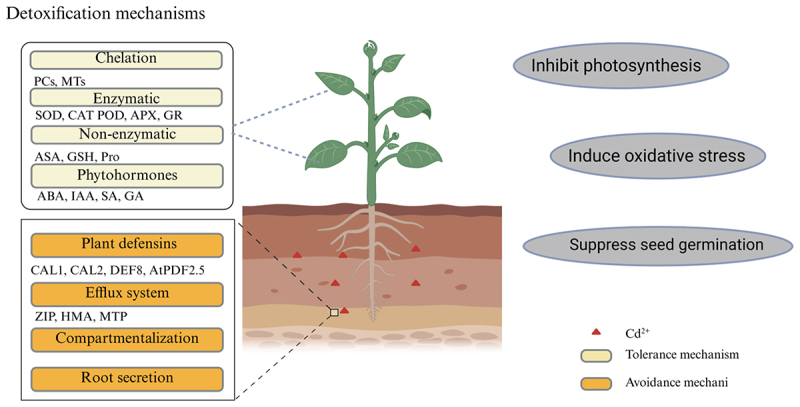
Plant response mechanisms under cadmium stress. MTs (metallothioneins); SOD (superoxide dismutase); POD (peroxidase); CAT (catalase); GSH (glutathione); PCs (phytochelatins); APX (ascorbate peroxidase); GR (glutathione reductase); ASA (ascorbic acid); pro (proline); ABA (abscisic acid); IAA (growth hormone); SA (salicylic acid); GA (gibberellic acid); ZIP (Zn-regulated transporter); HMA (heavy metal ATPase); MTP (metal tolerance protein). (mapping with BioRender).

Plants deploy multilayered detoxification systems to counteract cadmium toxicity, operating through two principal strategic frameworks (Figure 2). The avoidance mechanism involves root secretions that influence heavy metal movement,^59^ compartmentalization of Cd ions in cell walls and vesicles, and metal efflux.^60^ The tolerance mechanism enables plants to mitigate Cd^2+^ toxicity through chelation, antioxidant systems, phytohormone, and transporter proteins.^61–63^

#### 4.2.1. Avoidance mechanisms

##### 4.2.1.1. Root secretion

Plants mitigate heavy metal toxicity by secreting organic acids and other substances from their root systems, which alter metal bioavailability and promote microbial activities for synergistic detoxification.^64^ Under Cd stress, plant roots release various organic acids, including citric acid, oxalic acid, malic acid, tartaric acid, and succinic acid.^65^ These organic acids chelate ions to form stable, nontoxic complexes, and alleviate Cd toxicity through promoting plant growth and enhancing antioxidant enzyme activity.^66,67^ The specific organic acids released and their role in detoxification vary among plant species. In *Zea mays* L., root Cd content was positively correlated with the secretion of acetic acid, oxalic acid, and glutamic acid.^65^ Zhu et al.^68^ reported that under Cd treatment, tomatoes promoted root secretion of oxalic and citric acids, with oxalate helping to mobilize Cd^2+^ into the root system and improving Cd resistance. Sunflower showed a significant decrease in citrate levels under Cd stress, while malate and acetic acid levels increased.^69^ In *Ipomoea batatas* L., two ecotypes showed significant differences in Cd accumulation, which was closely related to the organic acids secreted by their roots. Notably, low Cd-accumulating varieties had a greater capacity for organic acid secretion than high Cd-accumulating types.^70^ Xin et al.^71^ found that under high Cd (10 µmol/L) treatment, low Cd varieties of pepper excreted significantly less tartaric acid and more oxalic and acetic acids compared to high Cd varieties, with no differences in citric or succinic acid levels between the two.

##### 4.2.1.2. Compartmentalization

Plant cell walls and vacuoles play a key role in compartmentalizing Cd, mitigating its toxicity.^72^ Vacuolar compartmentalization varies across plant species, with some efficiently sequestering ions in the vesicles for transport to aboveground parts.^73^ Vacuolar compartmentalization is a key process in plant cells. It partitions cellular components through the formation of vesicles and is facilitated by vesicle trafficking and transport processes. These processes are regulated by membrane transporter proteins and ion channels, which are crucial for cell homeostasis, growth, and development.^74^ In Cd-enriched *Sky Blue Zephyr* L., Cd content in epidermal cells was linearly correlated with cell length, indicating vesiculation promotes Cd^2+^ accumulation.^75^ In *Solanum nigrum* L., about 60% of Cd^2+^ was immobilized in the cell wall under Cd stress, with increased pectin methyl esterase (PME) activity promoting the exposure of negatively charged sites in the cell wall and Cd^2+^ adsorption.^76^ Similarly, in *Oryza sativa* L., X-ray Absorption Near-edge Structure (XANES) analysis showed Cd complexes formed primarily with the hydroxyl groups of hemicellulose in the root cell wall, and silicon enhanced lignification, reducing Cd^2+^ translocation.^77^ In *Zea mays* L. and *Arabidopsis thaliana* L., Cd detoxification is largely attributed to vesicular compartmentalization^78,79^ of key metal tolerance proteins (MTPs) in vesicular membranes regulate ion homeostasis, and overexpression of AtHMA3 transporters in tobacco increased a 3-fold vesicular Cd accumulation without affecting biomass, demonstrating, highlighting vesicular compartmentalization as a viable remediation strategy.^80^ However, when external metal concentrations exceed a threshold, the cell wall’s binding capacity is overwhelmed, allowing metal ions to penetrate the cytoplasm and organelles, causing toxicity.^81^ Vesicles and cytoplasm contain proteins, polysaccharides, organic acids and bases that form stable complexes with metals, thus reducing their bioavailability and toxicity. This may be the reason why Cd^2+^ is mainly concentrated in the vacuoles and cytoplasm.^72^

##### 4.2.1.3. Efflux systems

Plants utilize specialized transporters to extrude Cd^2+^ from the cytoplasm or compartmentalize it into inert subcellular regions, these transporters are like tiny molecular workers, precisely carrying out their tasks.^82^ By removing Cd^2+^ from the cytoplasm, plants prevent its harmful effects on essential cellular processes. Compartmentalization is like a strategic storage method, safely isolating toxic ions away from sensitive cellular components. This complex process constitutes critical detoxification pathways.^83^ The diversity of Cd^2+^ exocytosis systems, including HMAs, MTPs, and ABCs, offers molecular targets for low-Cd plant breeding. Heavy metal transporter ATPases (HMAs) belong to the P₁B-ATPase family, which is a well-defined group of proteins with distinct structural and functional characteristics. This family has evolved to perform the crucial task of heavy metal transport, which uses ATP hydrolysis energy to drive Cd translocation across membranes. *Arabidopsis thaliana* L. *AtHMA4* overexpression reduces shoot Cd accumulation by 50% through enhanced xylem loading and root-to-soil export.^84^ Metal-tolerant proteins (MTPs) function in a highly specific manner, with their unique structures allowing them to interact selectively with Cd ions. Pump Cd into the vesicle or secrete it extracellularly via a reverse proton transporter. Vesicular *AtMTP3* facilitates Cd^2+^/H^+^ antiport, achieving threefold higher vacuolar Cd concentrations and doubling biomass under 100 µmol/L Cd^2+^ stress versus wild-type.^85^ ABC transporter proteins (e.g., AtPDR8) enhanced tolerance by exocytosis of Cd chelates (e.g., PC-Cd), this process involves the formation of stable complexes between the transporter, the chelate, and the Cd ion, ensuring efficient removal of the toxic substance from the cell. While vesicular *ScYCF1* in yeast sequesters GSH-Cd conjugates, with knockout strains showing 80% growth inhibition under Cd exposure.^86^

#### 4.2.2. Tolerance mechanisms

##### 4.2.2.1. Chelation and sequestration

Plant chelating peptides (PCs) and metallothioneins (MTs) are key detoxification systems for plants to cope with Cd stress. These systems are like guardians, constantly protecting plant cells from the toxic effects of Cd. PCs form complexes with Cd, guiding it into vesicles for safe storage, and MTs bind to Cd tightly and also help fend off oxidative damage caused by Cd stress. PCs primarily facilitate vesicular compartmentalization, while MTs contribute additional metal-binding and antioxidant functions.^87^ Their synergistic action offers an important strategy for phytoremediation of Cd-contaminated soils, and genetic engineering and microbial interactions may further optimize their detoxification potential.^88^

PCs, synthesized from glutathione via phytochelatin synthase (PCS), form stable Cd^2+^-S complexes through thiol coordination. These complexes are subsequently sequestered into vacuoles via ABCC transporters, effectively reducing cytosolic Cd^2+^ concentrations.^89^ In *Oryza sativa* L., the expression of *OsPCS1* in low-Cd accumulating varieties was significantly higher than in high-Cd varieties, and higher PC levels correlated with greater Cd retention in the roots; Knockdown of *OsPCS1* by CRISPR-Cas9 led to decreased Cd tolerance, suggesting its critical role in Cd detoxification.^90^ In *Arabidopsis thaliana* L., *AtPCS1* gene mutant (*cad1–3*) had a complete deletion of PC synthesis under Cd stress, resulting in plants that were highly sensitive to Cd, while transgenic lines overexpressing *AtPCS1* had a twofold increase in vesicular Cd accumulation.^91^

MTs provide Cd detoxification through cysteine (Cys)-rich domains^92^ and are classified into four isoforms. Type 1 MTs are mainly involved in Zn/Cu homeostasis but also respond to Cd stress. Tobacco *NtMT1* expression was upregulated under Cd stress, promoting Cd^2+^ accumulation in roots and reducing its translocation to leaves, along with increased chlorophyll content, indicating enhanced antioxidant capacity.^93^ Type 2 MTs, with a higher Cd^2+^ affinity due to enriched Cys–Cys motifs, play a key role in Cd compartmentalization or exocytosis, as demonstrated in *Arabidopsis thaliana* L. and soybean.^94–96^

In addition, plant defensins play significant roles in cytoplasmic Cd detoxification and trafficking. Molecular studies in *Arabidopsis thaliana* L. and *Oryza sativa* L. have identified key defensin genes, such as *CAL1, CAL2, DEFENSIN 8* (*DEF8*), *AtPDF2.5, AtPDF2.6*.^97^ In *Arabidopsis thaliana* L., *AtPDF2.5* promotes cytoplasmic Cd^2+^ efflux through chelation, enhancing Cd detoxification and apoplastic accumulation, while *AtPDF2.6* detoxifies cytoplasmic Cd^2+^ through chelation, improving Cd tolerance.^98,99^ Distinctly, *CAL1* and *CAL2* typically function in the root cell wall. This has been demonstrated in *Arabidopsis thaliana* L. and *Oryza sativa* L., where *CAL1* and *CAL2* bind to Cd^2+^ in the cytoplasm; these complexes are then secreted into the apoplast of xylem parenchyma cells, facilitating Cd^2+^ translocation to the shoots.^100,101^ In *Oryza sativa* L., Cd-exposed seedlings produce *DEF8*, a dual-function protein that binds Cd^2+^ and facilitates its movement into phloem, thereby reducing Cd accumulation in grains.^102^

##### 4.2.2.2. Antioxidant systems

The plant antioxidant system combats Cd-triggered reactive oxygen species (ROS) through enzymatic and non-enzymatic components.^103^ These components work in harmony, with the enzymatic ones acting as catalysts to break down ROS, and the non-enzymatic ones directly scavenging or binding to ROS to neutralize their harmful effects. Enzymatic defenses include superoxide dismutase (SOD), catalase (CAT), peroxidase (POD), ascorbate peroxidase (APX), and glutathione reductase (GR), while non-enzymatic mechanisms involve (AsA), glutathione (GSH), metallothioneins (MTs) and proline (Pro).^67^

Under Cd stress, plants activate antioxidant defenses, but excessive exposure can impair this system, leading to its dysfunction and oxidative damage.^103^ After 7 days of 150 µmol/L CdCl_2_ treatment in wheat seedlings, SOD, CAT, APX, and GR increased initially (days 1–3), followed by progressive enzyme inactivation and 3.5-fold MDA accumulation, indicating lipid peroxidation.^104^ Similar patterns occur in *Solanum lycopersicum* L.,^105^ *Oryza sativa* L.,^106^ *Zea mays* L.,^107^ *Brassica napus* L.,^108^ where Cd stress exceeded the tolerance threshold, causing antioxidant system irreversible damage.

Central to non-enzymatic defense, the AsA-GSH cycle dynamically regulates redox homeostasis by scavenging ROS and regenerating oxidized glutathione (GSSG), thereby reducing Cd^2+^ toxicity.^109^ In *Brassica juncea* L., 50 µmol/L CdCl₂ elevated AsA levels 121% (2.8 mg/g FW (control) to 6.2 mg/g FW) and dehydroascorbic acid reductase (DHAR) activity 3.1-fold within 72 h.^110^ *Zea mays* L. roots under 100 µmol/L Cd^2+^ stress expanded total glutathione pools 2.8-fold but collapsed GSH/GSSG ratios from 4.7 to 1.3 within 48 h, signaling redox buffer saturation.^111^ These compensatory mechanisms fail when Cd exceeds species-specific thresholds, triggering AsA/GSH synthesis inhibition and irreversible oxidative damage.^112^ Thus, enhancing the synthesis of ASA and GSH may be a promising strategy for improving Cd tolerance in plants.

##### 4.2.2.3. Phytohormone signalling networks

Phytohormones act as central orchestrators of Cd^2+^ detoxification, integrating perception signals into physiological responses. They regulate antioxidant enzyme systems, activate specific transcription factor networks, and modulate heavy metal uptake and partitioning.^113^ Endogenous hormone dynamics reflect this regulatory role: in wheat under Cd stress (3 mg/kg), levels of abscisic acid (ABA), auxin (IAA), and gibberellins (GA) exhibit transient increases followed by declines, signifying active stress adaptation.^114^ Exogenous applications further demonstrate key functions. In *Hordeum vulgare* L., SA mitigates Cd-induced IAA depletion in root tips, reduces peroxidative damage and ROS accumulation, enhances antioxidant defenses, and maintains cellular osmotic balance and membrane integrity.^115^ Schellingen et al.^116^ demonstrated that the ethylene signaling pathway critically regulates the oxidative stress response to Cd in *Arabidopsis thaliana* L. Studies were shown that exogenous ABA enhances *Triticum aestivum* L. seedling tolerance to Cd by boosting antioxidant enzyme activity and proline content, counteracting growth inhibition.^117^ Furthermore, Cd stress signaling interacts with nitrogen-containing compounds like nitric oxide (NO), spermine (Spm), and spermidine (Spd).^118^ For instance, exogenous Spm/Spd treatment enhances *Triticum aestivum* L. Cd resistance by inhibiting lipid peroxidation, elevating glutathione content and reductase activity, and activating the antioxidant cycle.^119^

## 5. Multi-omics insights into plant Cd stress adaption

Integrative multi-omics approaches have revolutionized the systematic dissection of plant responses to Cd stress (Table 1), elucidating coordinated regulation across genetic, metabolic, and proteomic networks.^120^ Genome-wide association studies (GWAS) in *Ricinus communis* L. identified 181 SNPs associated with Cd tolerance, pointing *LOC8279875* as a key regulator of Cd transport.^121^ Transcriptomic profiling of *Nicotiana tabacum* L. under Cd exposure revealed 3232 up-regulated differentially expressed genes (DEGs) and 3278 down-regulated DEGs, including three WRKY transcription factors that suppress Cd accumulation through transcriptional reprogramming.^122^ Proteomics in *Populus yunnanensis* L. showed *MAPK3/6* (mitogen-activated protein kinase 3/6) and *CCOAOMT* (caffeoyl-CoA O-methyltransferase) enhance Cd compartmentalization.^123^ In *Arabidopsis thaliana* L., transcriptomic studies revealed extensive transcriptional changes, involving alterations in the expression of genes associated with signaling pathways and metabolic processes, which are essential for the plant’s adaptation to Cd stress.^124^ In *Oryza sativa* L., coordinated expression of Cd transporter proteins (*Nramp1, Nramp5, IRT1, HMA3)* was observed at both transcript and protein levels through transcriptomics, proteomics, and metabolomics.^125^

**Table 1.** Multi-omics insights into plant responses to cadmium stress.

Plant species	Methods	Results
*Ricinus communis* L.^121^	GWAS	*LOC8279875* regulates Cd transport
*Nicotiana tabacum* L.^122^	Transcriptomics	*WRKY12/27/33* suppress Cd accumulation
*Populus yunnanensis* L.^123^	Proteomics	Upregulation of *MAPK3/6* and CCOAOMT enhanced Cd compartmentalization.
*Arabidopsis thaliana* L.^124^	Transcriptomics	Altered expression of WRKY *TFs* and glutathione metabolism genes under Cd stress.
*Oryza sativa* L.^125^	Multi-omics	Coordinated expression of *Nramp1*, *HMA3*, and *IRT1*

These findings collectively highlight three core adaptive mechanisms: (1) transporter-mediated ion homeostasis (*HMA3, Nramp1*), (2) phytochelatin-based chelation networks, and (3) enzymatic/non-enzymatic antioxidant cascades. Future investigations could integrate single-cell spatial transcriptomics with epigenetic profiling to resolve tissue-specific stress adaptation mechanisms and provide new strategies for breeding crops for Cd tolerance.^126^

## 6. Conclusions

Current research has elucidated key plant adaptive mechanisms to Cd stress, including rhizospheric modulation via organic acid exudates, cellular compartmentalization (vacuole and cell wall) mediated by ABCC transporters and HMA ATPases, and thiol-based chelation through phytochelatins (PCs) and metallothioneins (MTs). The integrated antioxidant system, comprising enzymatic components (SOD, CAT, APX) and the AsA-GSH cycle, demonstrates dose-dependent efficacy in neutralizing Cd-induced ROS. These mechanisms enable hyperaccumulators to tolerate elevated Cd concentrations while maintaining physiological functions.

Despite identifying critical transporters (e.g., ZIP, NRAMP, HMA) and chelators, their regulatory networks across developmental stages and environmental gradients remain incompletely characterized. Future efforts should prioritize multi-omics integration to dissect Cd sensing pathways, validate species-specific tolerance thresholds, and develop modular phytoremediation platforms combining transporter/chelator engineering (e.g., PCs/MTs overexpression) with rhizosphere microbiome management. While multi-omics technologies have provided mechanistic insights, data integration faces challenges due to the heterogeneity in experimental designs (e.g., growth stages, Cd exposure regimes). Addressing these challenges will enable precision strategies of Cd-contaminated ecosystems, ensuring agricultural safety and environmental sustainability.
